# Intracellular pocket and ICL2 motifs govern G-protein coupling bias in NK_1_ receptor (TACR1) and NK_2_ receptor (TACR2)

**DOI:** 10.1016/j.jbc.2026.113437

**Published:** 2026-08-12

**Authors:** Jacob E. Petersen, Artem Pavlovskyi, Jesper J. Madsen, Thue W. Schwartz, Thomas M. Frimurer, Ole H. Olsen

**Affiliations:** 1 Novo Nordisk Foundation Center for Basic Metabolic Research, University of Copenhagen, Copenhagen N, Denmark; 2 Department of Molecular Medicine, Morsani College of Medicine, University of South Florida, Tampa, Florida, USA; 3 Center for Global Health and Infectious Diseases Research, Global and Planetary Health, College of Public Health, University of South Florida, Tampa, Florida, USA

**Keywords:** G-protein–coupled receptor (GPCR), G_q_ and G_s_ signaling, biased signaling, molecular dynamics, molecular modeling

## Abstract

The tachykinin receptors NK_1_ receptor (TACR1) (NK1R) and NK_2_ receptor (TACR2) (NK2R) are G-protein–coupled receptors that preferentially bind substance P (SP) and neurokinin A (NKA), respectively. Although these ligands differ in their N-terminal sequences, a conserved C-terminal motif confers partial cross-reactivity. In COS-7 cells, both NK1R/SP and NK2R/NKA activate G_q_ and G_s_ signaling pathways to a certain extent. NK1R/SP displays an approximately threefold preference for G_s_ coupling, whereas NK2R/NKA couples comparably to G_q_ and G_s_, albeit with lower overall potency. In contrast, NK1R/NKA preferentially activates G_q_, while NK2R/SP exhibits negligible activity. Receptor activation enables insertion of the C-terminal segment of the Gα α5-helix (so-called “wavy-hook”) into the intracellular receptor pocket, involving intracellular loop 2. We investigate the structural basis for differential G_q_ and G_s_ coupling by NK1R and NK2R by SP and NKA. Analysis of structures identifies four key differences between NK1R and NK2R located at the base of the intracellular pocket and within intracellular loop 2. Reciprocal mutagenesis swapping the motifs between NK1R and NK2R demonstrates that alterations in this region can redirect G-protein coupling and signaling bias.

The neurokinin receptor (NKR) family (1, 2) and its peptide ligands, collectively known as tachykinins, are of considerable interest as established and potential drug targets. Within the rhodopsin-like class A of the G-protein–coupled receptor (GPCR) superfamily, the NKR family comprises three members: NK_1_ receptor (TACR1) (NK1R), NK_2_ receptor (TACR2) (NK2R), and NK_3_ receptor (TACR3) (NK3R). Each receptor is preferentially activated by a high-affinity endogenous peptide—substance P (SP) for NK1R, neurokinin A (NKA) for NK2R, and neurokinin B (NKB) for NK3R. Although these ligands differ in their N-terminal sequences, they share a conserved C-terminal motif that permits cross-activation of all three receptors. NKRs have been implicated in a wide range of pathological conditions, including ocular disorders (3), chemotherapy-induced nausea and vomiting (*via* approved NK1R antagonists), anxiety disorders and asthma (4, 5, 6, 7) (*via* NK2R antagonists), and disorders such as schizophrenia, hypertension, reproductive disorders, and preeclampsia through NK3R (7). In addition, NKRs are under investigation as therapeutic targets for obesity and related metabolic disorders (8, 9, 10, 11, 12). In particular, recent studies demonstrate that long-acting NK2R agonists can significantly reduce body weight, blood glucose, triglycerides, and cholesterol levels while improving insulin sensitivity in diabetic, obese nonhuman primates (13, 14).

Recent years have seen significant advances in structural knowledge of NKRs, with X-ray crystallography of NK1R structures in complex with various antagonists (15, 16, 17), paving the way for conformational ensemble studies (18, 19, 20) and new functional insights. In addition, cryo-EM has resolved the structures of NK1R bound to SP and signal mediators G_q_ and G_s_, NK2R bound to NKA and G_q_, as well as NK3R in complex with SP, NKB, senktide, and G_q_ (6, 7, 21, 22, 23). Interestingly, in the cryo-EM structures of NK1R bound to SP with either G_q_ or G_s_, both G-proteins adopt nearly identical conformations relative to NK1R, the only notable difference being the position of the Cα5 helix of G_q_, which sits deeper than that of G_s_ (22), suggesting no preferential coupling of G_q_ or G_s_ to NK1R. However, several studies have reported biased signaling in NK1R (23, 24).

We have extensively explored the activation mechanism of NK1R and NK2R using molecular dynamics (MD) simulations and mutagenesis (25). These studies identified a phenylalanine in NK1R and a tyrosine in NK2R at position 6.51 (Ballesteros–Weinstein numbering (26)) in transmembrane helix 6 (TM6), adjacent to a conserved serine in TM7 (7.45) and the CWxP microswitch motif. MD simulations have shown that rotation of the CWxP tryptophan facilitates communication with the NPxxY microswitch motif in TM7. Accordingly, the conserved serine at position 7.45 was proposed to act as a central hub in NKR activation. Furthermore, we have previously investigated (27) the effects of truncated SP and NKA analogs on signaling activity. Progressive truncation of SP and NKA (SP(6–11) and NKA(6–10)) revealed ligand-dependent coupling preferences: NK1R activated by SP analogs preferentially engaged G_s_ over G_q_, whereas activation by NKA analogs resulted in G_q_ potencies nearly an order of magnitude higher than those for G_s_. In contrast, NK2R activated by NKA analogs displayed only minor differences between G_q_ and G_s_ potencies. The N-terminal charge status plays a key role, leading to significant differences in analog signaling potency.

In the present work, we consider the intracellular region of NK1R and NK2R to investigate how perturbations at the interface between NKRs and the G-protein influence coupling and signaling bias (28, 29). The NKR/G-protein interface can be conceptually divided into two cooperative but functionally distinct interaction modules: an intracellular loop 2 (ICL2)–driven receptor interface and a G-protein–mediated interface formed by the C-terminal segment of the Gα α5 helix, referred to as the “wavy-hook” (30). Upon receptor activation, ICL2 typically adopts a short, often strained α-helical conformation that projects into the cytoplasmic cavity, whereas the wavy-hook inserts deeply into the activated receptor core formed by transmembrane helices TM3, TM5, TM6, and associated intracellular loops. The roles of individual residues in ICL2 for primary and secondary GPCR-G-protein coupling have been discussed (31). - The article is structured as follows. First, ICL2 module is considered. Sequence analysis reveals that ICL2 of NKRs is unusually short (six residues) and exhibits a sequence unique among class A GPCRs: NK1(2)R-137(138):PL(F)Q(K)PRL (residues in parentheses denote NK2R). Mutagenesis and MD simulations demonstrate that proline residues (particularly P137 (138)) are critical for receptor activity, while inspection of cryo-EM structures shows that L(F) directly contacts the Gα subunit and that the arginine anchors TM2, TM3 and TM4 through a compact hydrogen bond (Hbond) network. Swapping the L(F) position between NK1R and NK2R induces pronounced alterations in G_q_ and G_s_ signaling. Next, residues forming the wavy–hook interaction module were examined. Introduction of individual NK2R residues into NK1R produced divergent functional outcomes: one substitution markedly enhanced G_q_-biased signaling by both SP and NKA, increasing potency and efficacy with minimal effects on G_s_ signaling, whereas another substitution conferred signaling bias by selectively impairing G_s_ coupling while largely preserving G_q_ activity. Conversely, introduction of NK1R residues into NK2R enhanced both G_q_ and G_s_ signaling. Finally, combining mutations across the two functional modules demonstrates their coorporative role as a two-part molecular handshake governing G-protein selectivity.

## Results and discussions

**Figure 1:**
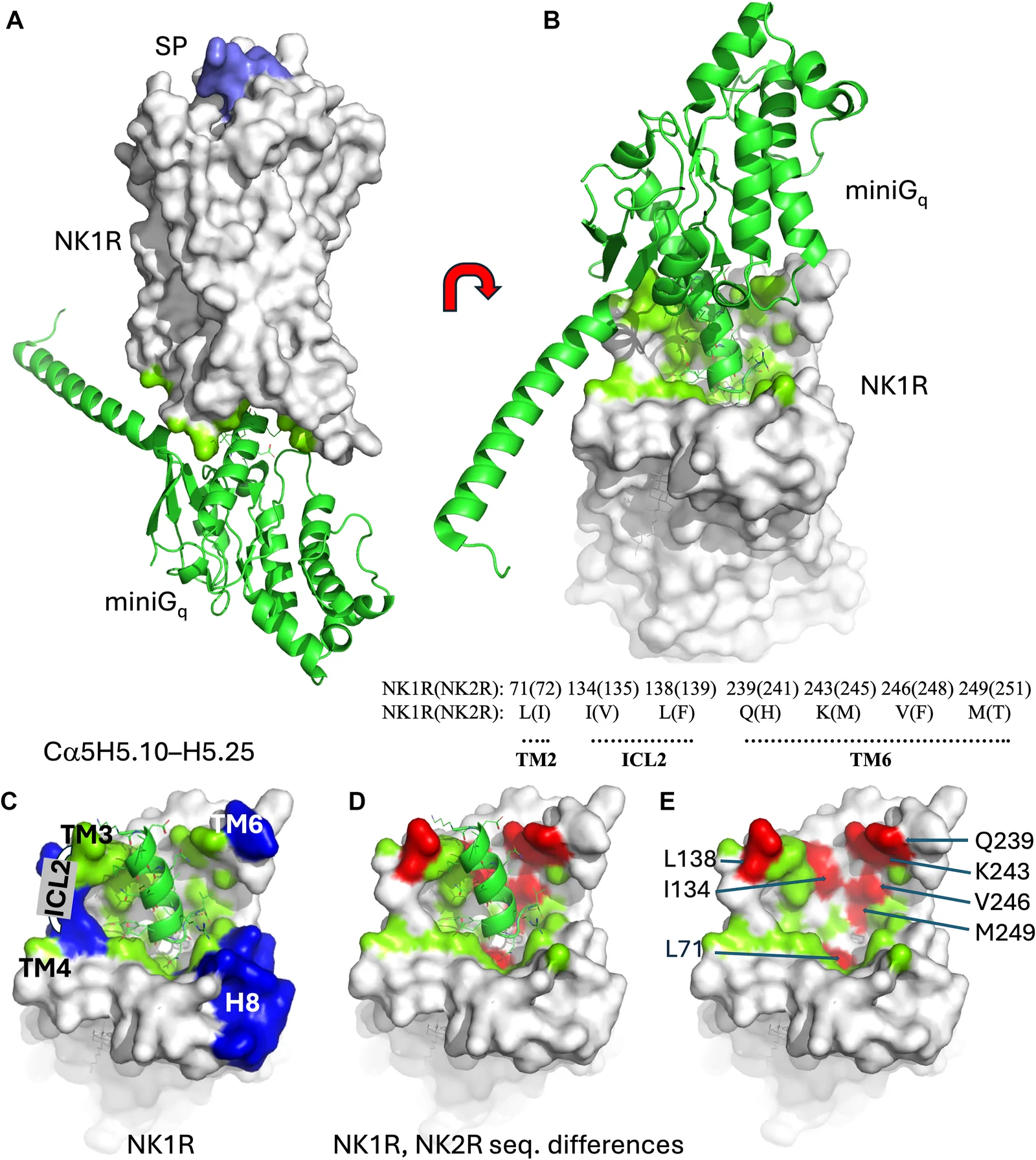
**Structure of the NK1R–SP–miniG_q_ complex.***A*, overall view of the NK1R/SP/miniG_q_ complex (PDB entry: 7p00). NK1R is shown as a *gray surface*, SP as a *light blue surface*, and miniG_q_ as a *green cartoon*. NK1R atoms within 4 Å of miniG_q_ are highlighted in *light green*. *B*, the complex shown in (*A*) rotated by 180° to illustrate the NK1R/miniG_q_ interaction interface. *C*, surface representation of NK1R as in (*B*), with the miniG_q_ α5 helix (residues Gα H5.10–H5.25, (the *wavy-hook*) shown in cartoon representation. ICL2 (connecting TM3 and TM4), the termini of TM3, TM4, and TM6 (*blue*), as well as H8 (*blue*) have been labeled. *D*, side-chain differences between NK1R and NK2R mapped onto NK1R and highlighted in *red*. *E*, α5 helix removed. Specification of the side chain substitutions distinguishing NK1R from NK2R (in parentheses) in TM2: L71(I72)^2.43^, in TM3: I134(V135)^3.54^, ICL2: L138(F139)^34.51^, and in TM6: Q239(H241^)6.26^, K243(M245)^6.30^, V246(F248)^6.33^, and M249(T251)^6.36^. ICL2, intracellular loop 2; NK1R, NK1 receptor; NK2R, NK2 receptor; SP, substance P; TM, transmembrane.

To provide structural context Figure 1, *A* and *B* present overall views of the cryo-EM structure of the NK1R/SP/miniG_q_ complex (PDB: 7P00, (22)). NK1R is displayed as a gray surface, SP as a blue surface, and miniGq as a green cartoon, with NK1R residues within 4 Å of miniG_q_ highlighted in light green. In Figure 1*B*, the complex is rotated by 180° to emphasize the NK1R–miniG_q_ interaction interface. Figure 1*C* presents a surface representation of NK1R with the miniG_q_ Ga α5 helix (residues H5.10–H5.25, the wavy-hook) shown in cartoon representation. For clarity and orientation, key regions of the receptor surface have been highlighted in blue and labeled. Specifically, ICL2, shown in blue and connecting TM3 and TM4 (with the termini labeled), and the N-terminal region of TM6 in blue have been highlighted. In addition, H8, the short amphipathic helix located immediately after TM7 at the intracellular C terminus of the receptor, has been highlighted in blue and labeled. Sequence comparison reveals remarkably few differences between NK1R and NK2R within this interface. These differences are mapped onto NK1R in Figure 1*D* and highlighted in red. For clarity, the α5 helix is removed in Figure 1*E*, which illustrates the side chain substitutions distinguishing NK1R from NK2R (NK2R residue numbers in parentheses) in TM2: L71(I72)^2.43^, in TM3: I134(V135)^3.54^, in ICL2: L138(F139)^3.51^, and in TM6: Q239(H241)^6.26^, K243(M245)^6.30^, V246(F248)^6.33^, M249(T251)^6.36^. Reciprocal mutagenesis swapping these residues between NK1R and NK2R demonstrates that alterations in intracellular pocket chemistry can reprogram G-protein coupling, as quantified by bioluminescence resonance energy transfer (BRET)-based cAMP (G_s_) and IP_3_ accumulation (G_q_) signaling assays.

Although the present study focuses on the interface between NK1R and NK2R and their interactions with G_s_ and G_q_, it is noteworthy that NK1R and NK3R exhibit a high degree of sequence conservation. In particular, the intracellular positions identified here as determinants of G-protein coupling are identical in NK1R and NK3R. This conservation suggests that the mechanistic insights gained from the NK1R mutagenesis are likely to extend to NK3R as well.

### NKR’s ICL2 module

**Figure 2:**
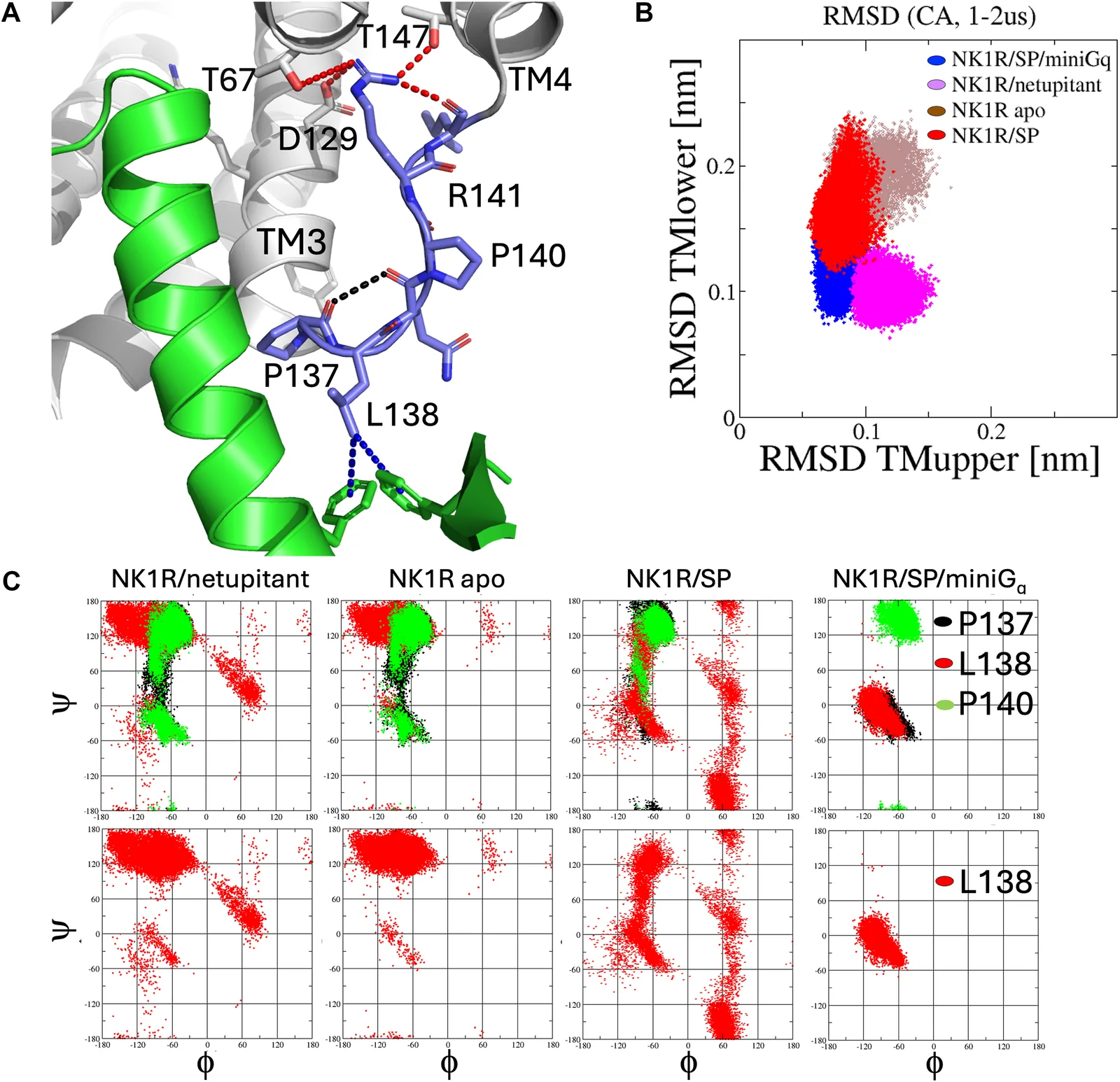
**ICL2 structure, sequence analysis and MD simulation results.***A*, the intracellular loop 2 (ICL2; NK1R residues 137–142, sequence PLQPRL) from the NK1R/SP/miniG_q_ complex (PDB: 7P00) is shown as a *blue stick model* connecting transmembrane helices TM3 and TM4. Aromatic side chains from miniG_q_ (*green stick model*), located in the Gα α5 helix (*green cartoon*) and the β strand (*green cartoon*), form tight hydrophobic interactions (*blue dotted lines*) with NK1R-L138. NK1R-R141 (located in ICL2) forms a stabilizing salt bridge (*red dotted line*) with NK1R-D129^3.49^ (in TM3). NK1R-T67^2.39^ (in TM2), NK1R-T147^4.42^ (in TM4) and backbone carbonyl of NK1R-L142 form Hbonds (*red dotted lines*) with NK1R-R141 thereby linking TM2, TM3, TM4 and ICL2 together. The ICL2 loop adopts a strained conformation, as indicated by the unusually short distance (3.3 Å, *black dotted line*) between the backbone carbonyl oxygens of NK1R P137 and Q139. *B*, RMSD values for the four trajectories (from the time window 1-2 ms) are shown as pairwise plots of the *upper versus lower* halves of the TM bundle. NK1R/SP/miniG_q_ in *blue*, NK1R/netupitant in *magenta*, NK1R/SP in *red*, and NK1R apo in *brown*. *C*, ϕ/ψ distributions of ICL2 residues. Ramachandran maps of Pro137 (*black*), Leu138 (*red*), and Pro140 (*green*) from simulations of NK1R/netupitant, NK1R apo, NK1R/SP, and NK1R/SP/miniG_q_. The lower panel shows the ϕ/ψ distribution of Leu138. ICL2, intracellular loop 2; NK1R, NK1 receptor; SP, substance P; TM, transmembrane.

The ICL2 forms the primary receptor–side interaction surface for the G-protein (28, 32) and adopts upon receptor activation a strained loop (often α-helical) conformation that extrudes into the cytoplasmic cavity (Fig. 2*A*). This loop directly contacts the G-protein’s β2-β3 loop and the N-terminal portion of Gα α5 helix, facilitating a productive engagement. However, sequence variability within ICL2 of the receptor family translates into structural differences in the loop which in turn affect the interaction with the G-proteins. Especially the positioning of proline residues and the G-protein-facing L(F) position in ICL2 can restrain these interactions.

#### NKR’s ICL2 sequence is rare among class A GPCRs

A search of ICL2 sequences was performed based on available class A GPCR sequences (GPCRdb (33)). This analysis shows that most class A ICL2 loops have length 8, whereas only few are shorter or longer (Fig. S1*A*). NKRs are among the short outliers with a ICL2 sequence length of only 6. Curiously, the NKR ICL2 sequences carry a conserved sequence with two prolines, PxxP (specifically, PxxPRL). We note that only few class A GPCRs even carry this specific combination, ICL2 length of 6 and the PxxPxx motif (Fig. S1, *B* and *C*). In particular, the only class A GPCRs carrying this ICL2 architecture are the NKRs, neuropeptide FF receptor (peptide receptor (34)), prokineticin receptors 1 and 2 (protein receptor (35)), bile acid receptor GPBA (steroid receptor (36)), and GPR83 (orphan, implicated in metabolism (37))(Fig. S1*D*). We also note that extended analysis of class A GPCR ICL2 sequences indicates that ICL2 loops only very rarely contain poly-proline motifs at all, underscoring that the unique conformational restraints imposed by prolines and a short ICL2 loop give rise to distinct loop conformations. Taken together, these observations suggest that NKRs may have a unique ICL2 that affects engagement with the G-protein complex.

The ICL2 (NK1R residues 137–142, sequence PLQPRL) from the NK1R/SP/miniG_q_ complex (PDB: 7P00) is shown in Figure 2*A* as a blue stick model connecting TM3 and TM4. Aromatic side chains from miniG_q_ (green stick model), located in the Gα α5 helix (H5.08, conserved between G_q_ and G_s_) and the β2-β3 loop (miniG_q_ residue 212, also conserved between G_q_ and G_s_), engage in tight hydrophobic interactions with NK1R-L138. Distances between NK1R-L138 CD1 and the aromatic rings are indicated by blue dashed lines (4 Å and 5 Å). Within ICL2, NK1R-R141 forms a stabilizing salt bridge (red dotted line) with NK1R-D129^3.49^ in TM3, part of the conserved DRY microswitch motif. In addition, NK1R-T67^2.39^, NK1R-T147^4.42^ and backbone carbonyl of NK1R-L142 form Hbonds (red doted lines) with the guanidinium group of NK1R-R141, thereby linking TM2, TM3, TM4, and ICL2 into a tightly coupled network. The ICL2 loop adopts a strained conformation, as indicated by the unusually short distance (3.3 Å) between the backbone carbonyl oxygens of NK1R-Q137 and NK1R-P139 (black dotted line). A comparable ICL2 conformation is observed in the NK2R/NKA/miniG_q_ structure (PDB: 7XWO (6)) as well as in the NK3R/NKB/miniG_q_ structure (PDB: 8JBG (7)). However, in NK2R the conserved arginine within ICL2 bends back and forms a stabilizing Hbond with the main-chain carbonyl of a neighboring glutamine residue within the same loop, partially alleviating the conformational strain.

#### Conformational dynamics of ICL2

The strained conformation observed for ICL2 due to its PxxPRL motif motivated a detailed analysis of its conformational dynamics (38). To this end, MD simulations were performed on four receptor models: (i) NK1R in complex with the high-affinity antagonist netupitant, (PDB: 6HLP (17) – NK1R/netupitant), (ii) an apo NK1R model derived from 6HLP with netupitant removed (NK1R apo), (iii) an NK1R structure with SP bound (based on PDB: 7P00 with miniG_q_ removed – NK1R/SP), and (iv) the intact PDB ID:7P00 structure (NK1R/SP/miniG_q_). Fig. S2 shows RMSD values for the four trajectories, calculated separately for the upper and lower halves of the transmembrane (TM) bundle. After a brief equilibration period, all trajectories display stable behavior indicated by RMSD values. In the NK1R/netupitant trajectory, the upper and lower TM regions exhibit comparable deviations, whereas in the remaining simulations the upper bundle displays reduced flexibility relative to the lower bundle. These behaviors are illustrated in Figure 2*B*, which shows pairwise plots of RMSD values for the lower *versus* upper halves of the TM bundle, calculated from the 1-–2 μs time window for the four trajectories. The systems are color-coded as follows: NK1R/SP/miniG_q_ (blue), NK1R/netupitant (magenta), NK1R/SP (red), and NK1R apo (brown). The NK1R/SP/miniG_q_ complex exhibits the lowest RMSD values for both TM halves, whereas the NK1R apo system displays the highest RMSD values for both regions. The presence of the agonist SP favors lower RMSD values for the upper TM half, while the antagonist netupitant stabilizes both the upper and lower TM halves.

The MD-sampled dynamics of ICL2 were examined by analyzing the Ramachandran (ϕ/ψ dihedral angle) distributions of its constituent residues. Figure 2*C* presents Ramachandran plots for Pro137 (black), Leu138 (red), and Pro140 (green) derived from MD simulations of the NK1R/netupitant, NK1R apo, NK1R/SP, and NK1R/SP/miniG_q_ systems. The lower panel shows the Ramachandran plot of Leu138. Notably, the NK1R/SP/miniG_q_ trajectory stands out, as the conformational space sampled by all three residues is highly restricted and located within the most populated regions of experimentally observed Ramachandran maps (39). Consistent with this behavior, Leu138 and Pro137 predominantly adopt α-helical backbone conformations, whereas Pro140 favors a β-strand-like backbone conformation. In the NK1R/SP system, the presence of SP leads to intermittent sampling of α-helical backbone conformations, a behavior that is rarely observed in the NK1R/netupitant and NK1R apo systems (39) where the usual Ramachandran distribution of Pro137 and Pro140 is consistent with a variety of backbone conformations. Thus, ICL2 adopts a well-defined strained conformation in complex with SP and miniG_q_ induced by the prolines in positions 137 and 140.

To assess whether the observations from the NK1R MD simulations also apply to NK2R, additional simulations were performed on the NK2R/NKA and NK2R/NKA/miniG_q_ complexes (Fig. S3). These simulations revealed conformational behavior closely resembling that observed for the corresponding NK1R complexes, indicating that the underlying activation mechanism is conserved between the two receptors. Specifically, the Ramachandran distributions (Fig. S3) show that binding of NKA alone is insufficient to stabilize the active ICL2 conformation, whereas incorporation of miniG_q_ markedly restricts the conformational ensemble and stabilizes the active ICL2 geometry.

#### Mutagenesis of residues within the NK1R ICL2

**Figure 3:**
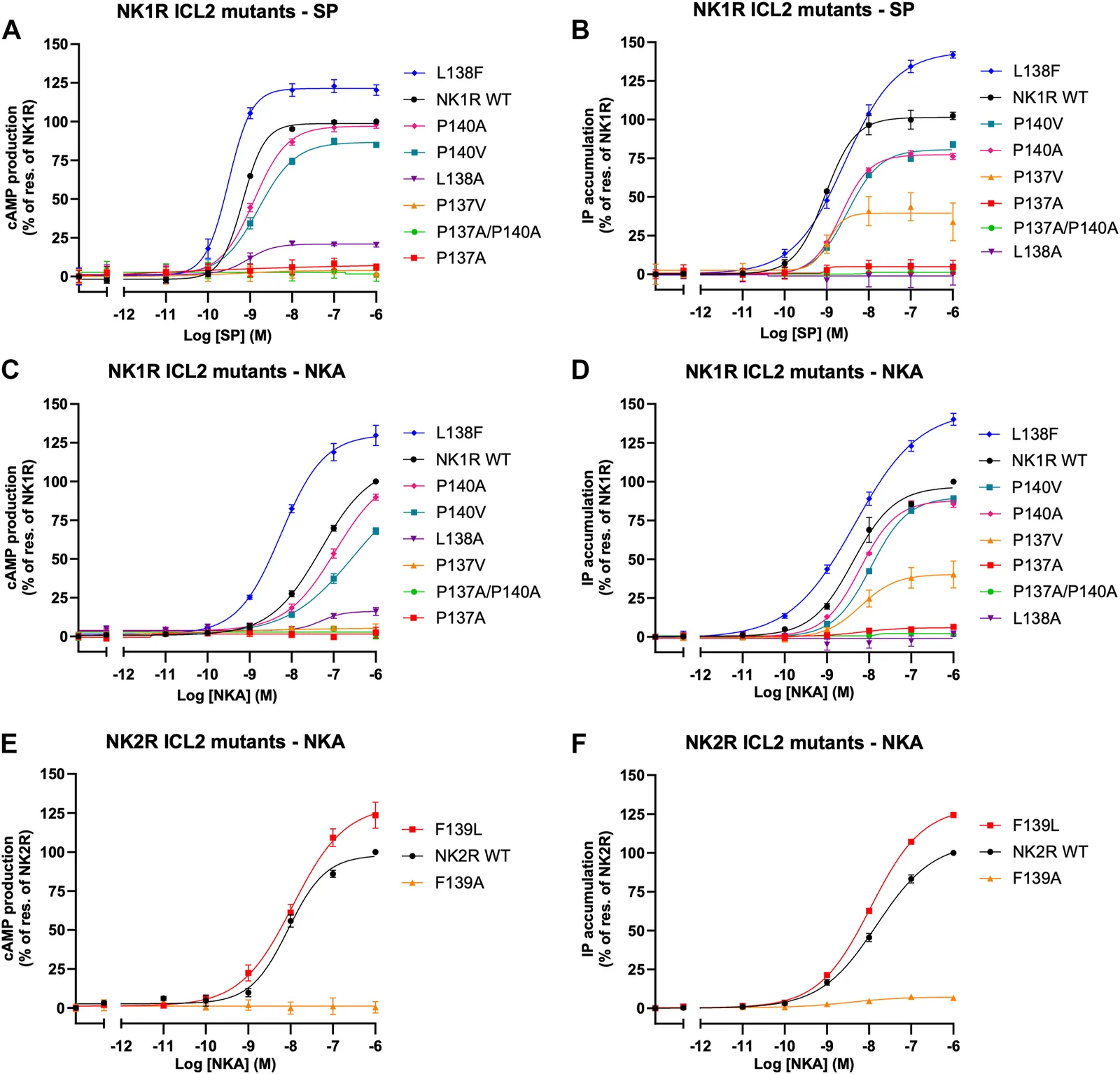
**Activation of NKR ICL2 mutations by SP and NKA.** BRET-based cAMP (G_s_) (*A*, *C*, and *E*) and IP_3_ accumulation (G_q_) (*B*, *D*, and *F*) responses to SP and NKA are shown. Mutations are specified to the right of the graphs in the order as the corresponding graphs. Dose–response values are presented as the mean of biological replicates, with error bars indicating the SEM. Error bars were not calculated for experiments with fewer than three biological replicates. Relevant data for each graph, numbers of biological and technical replicates are provided in Table S1. The leftmost data point in each graph corresponds to the vehicle control, *i.e.* an experiment performed in the absence of ligand. The NK1R-L138F (in *A*-*D*) mutant displays enhanced G_s_ signaling (both potency and efficacy), while all other mutants except P140A and P140V show minimal or negligible activity. A comparable pattern is observed for G_q_ signaling, except for SP stimulation (*B*), where NK1R-L138F retains NK1R-like potency but exhibits increased efficacy. (*E* and *F*) NK2R-F139L shows modest increase in potency and pronounced enhancement in efficacy. BRET, bioluminescence resonance energy transfer; NK1R, NK1 receptor; NKA, neurokinin A; NKR, neurokinin receptor; SP, substance P.

To test the proposed roles of the prolines and Leu138 (Phe139) in NK1R(NK2R) ICL2 in regulating receptor activity, these residues were mutated and their signaling properties were assessed using BRET-based cAMP (G_s_) and IP_3_ accumulation (G_q_) assays in response to both SP and NKA. In NK1R, the prolines were substituted with alanines to favor α-helical backbone conformations and with valine to promote β-strand–like conformations. The NK1R-L138F mutant was hypothesized *a priori* to enhance receptor activity, based on the observed tight interaction of Leu138 with two phenylalanine residues from the G-proteins (Fig. 2*A*), which could be further strengthened by aromatic–aromatic interactions. To further assess the functional importance of this position, Leu138 was also mutated to alanine. The NK2R-F139L mutant was expected *a priori* to exhibit reduced activity due to the loss of aromatic–aromatic interactions. Figure 3, *A*–*D* present the results of activity assays for NK1R ICL2 mutants. BRET-based cAMP (G_s_) responses to SP and NKA are shown in Figure 3, *A* and *C*, while IP_3_ accumulation (G_q_) responses are shown in Figure 3, *B* and *D*. ELISA assays (Fig. S4*A*) show that expression levels of NK1R and its mutants vary by only a few percent, indicating that differences in efficacy cannot be explained by differences in expression levels. The NK1R-L138A mutant exhibits no or negligible activity, underscoring the importance of this position. As predicted, the NK1R-L138F mutant exhibits enhanced signaling activity, reflected by increased potency and efficacy in three of the four assays (Fig. 3, *A*, *C*, and *D*). Specifically, G_s_ activation of the mutant is enhanced in response to both SP and NKA, and G_q_ activation is increased in response to NKA. In contrast, G_q_ activation by SP shows NK1R-like potency but improved efficacy (Fig. 3*B*). All other mutants except NK1R-P140A and NK1R-P140V show minimal activity. The observed increases in potency and efficacy of NK1R-L138F (Fig. 3, *A*, *C*, and *D*) indicate that the mutation stabilizes active receptor conformations and enhances productive G-protein coupling, shifting the conformational equilibrium toward signaling-competent states. The distinct effects of SP and NKA on G_q_ activation (Fig. 3, *B* and *D*) suggest that they stabilize different active conformations of NK1R, which differ primarily in their dependence on ICL2-mediated stabilization for productive G_q_ coupling. The complete loss of activity upon mutation of NK1R-Pro137 to Ala underscores its central role in stabilizing the ICL2 conformation. Together, the sequence analysis—revealing an unusually short loop with a distinctive proline motif—and MD simulations showing that NK1R-Leu138 adopts a productive Ramachandran conformation only in the NK1R/SP/miniG_q_ complex provide a rationale for the lack of constitutive activity in tachykinin receptors (24). In this view, NKRs maintain a strained ICL2 conformation that disfavors formation of the short canonical amphipathic α-helix typically associated with the active state of many GPCRs (40, 41). Consequently, the receptor must overcome a backbone strain penalty to adopt the active ICL2 geometry required for G-protein engagement.

#### Mutagenesis of residues within the NK2R ICL2

In NK2R, Phe139 was mutated to Leu and Ala. The prolines were not altered, as the high sequence homology of ICL2 among NKRs suggests a conserved role consistent with that observed in NK1R. Figure 3, *E* and *F* present the results of activity assays for the two NK2R ICL2 mutants, measured as BRET-based cAMP (G_s_) responses (Fig. 3*E*) and IP_3_ accumulation (G_q_) responses (Fig. 3*F*) upon stimulation with NKA. Experiments with SP were omitted due to its negligible activity in stimulating NK2R (21, 27). As observed for NK1R, ELISA assays (Fig. S4*B*) show that expression levels of NK2R and its mutants are comparable, indicating that differences in efficacy are not attributable to variations in expression levels. Consistent with expectations, the alanine mutant (NK2R-F139A) exhibited minimal activity in both assays, in agreement with the corresponding mutation in NK1R (Fig. 3, *A*–*D*). In contrast, the leucine mutant (NK2R-F139L) deviated from the *a priori* prediction, displaying a modest increase in potency and a more pronounced enhancement in efficacy. These results suggest that the mutation stabilizes active receptor conformations and promotes more productive G-protein coupling.

### NKR’s wavy-hook interface module

In Figure 1, *C* and *D*, the interaction between the Gα α5 helix and the NK1R is outlined, highlighting sequence differences at this interface between NK1R and NK2R. The relevant positions are located at TM2: L71(I72)^2.43^, TM6: V246(F248)^6.33^, M249(T251)^6.36^, for NK1R(NK2R). Results from reciprocal mutagenesis experiments, in which these residues were swapped among NK1R and NK2R, are discussed below.

**Figure 4:**
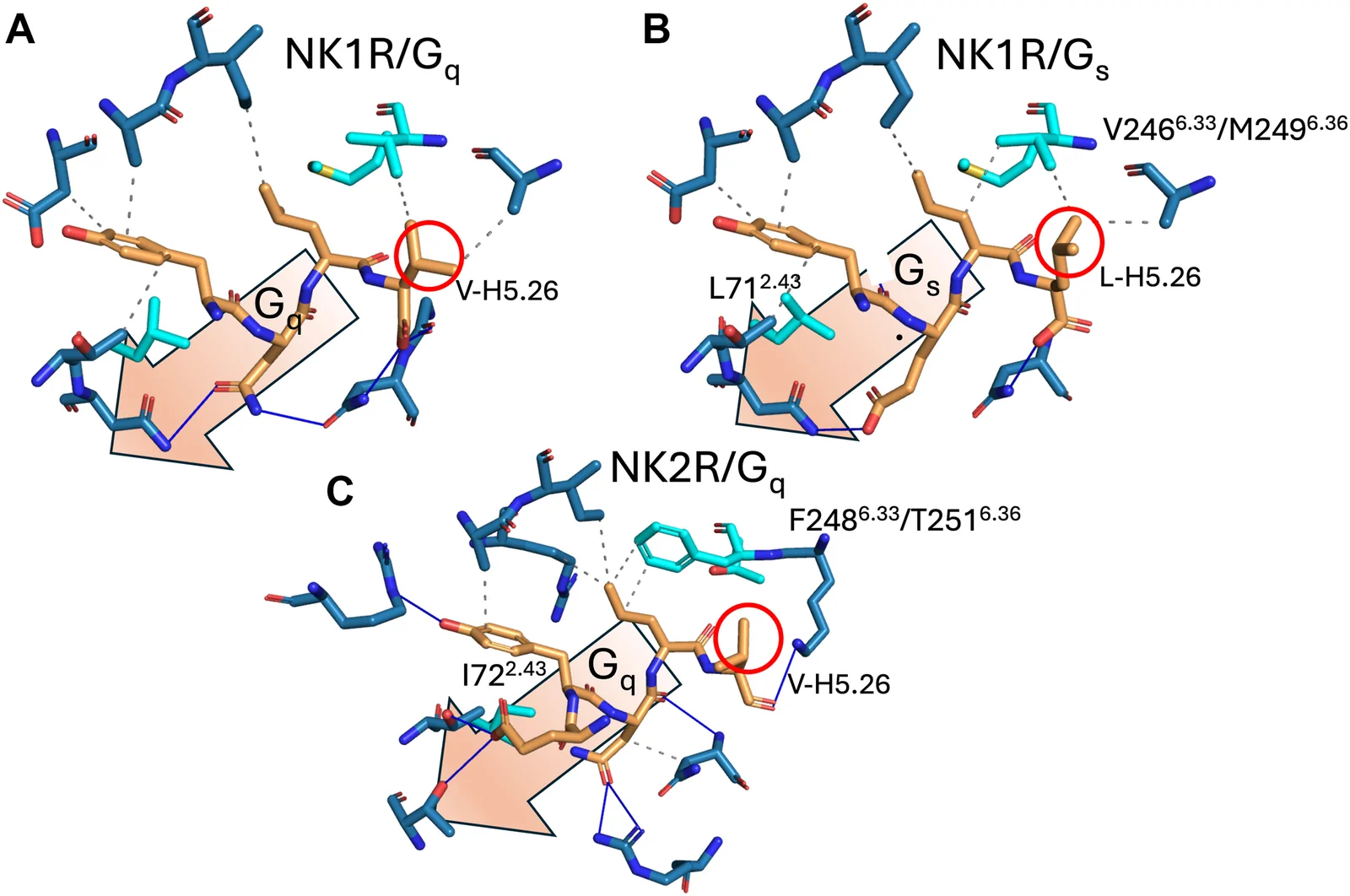
**Wavy-hook (H5.21–H5.26) interactions in NK1R and NK2R.** (*A*) NK1R/G_q_ (PDB: 7P00), (*B*) NK1R/G_s_ (PDB: 7P02), and (*C*) NK2R/G_q_ (PDB: 7XWO). G-proteins in *gold stick model* and, NK1R and NK2R in *blue stick models*, respectively. Hbonds in *solid lines* and hydrophobic interactions are represented by *dashed lines*. Side chains that differ between NK1R and NK2R are highlighted in *cyan*. The position of C-terminal α5 helix of Gα is indicated by *gold arrows* pointing toward its N-terminal direction and the C-terminal residue (Val in G_q_ and Leu in G_s_) is highlighted with *red circles*. NK1R, NK1 receptor; NK2R, NK2 receptor.

Figure 4 presents close-up views of interactions involving the C-terminal wavy-hook region (H5.21–H5.26) of the Gα α5 helix (the position of the helix is indicated by gold arrows pointing toward its N-terminal direction). Panels show (A) NK1R/G_q_ (PDB: 7P00), (B) NK1R/G_s_ (PDB: 7P02), and (C) NK2R/G_q_ (PDB: 7XWO). G-proteins are depicted as gold stick models, whereas NK1R and NK2R are shown as blue sticks. Hbonds are indicated by solid lines and hydrophobic interactions by dashed lines. Side chains that differ between NK1R and NK2R are highlighted in cyan, and the C-terminal residues of the G-proteins (Val in G_q_ and Leu in G_s_) are marked by red circles. Notably, the interaction patterns are strikingly similar (22).

The impact of single point mutations at TM2: 71(72)^2.43^, TM6: 246(248)^6.33^ and 249(251)^6.36^ as well as combination mutants (246(248)^6.33^/249(251)^6.36^ and 71(72)^2.43^/246(248)^6.33^/249(251)^6.36^ are evaluated. The combined mutations were designed to probe the effect of more extensive changes in surface chemistry on activity. ELISA assays (Fig. S4*A*) show that expression levels of NK1R and its mutants are comparable, indicating that differences in efficacy are not attributable to variations in expression levels.

#### Mutagenesis of residues at the NK1R/wavy-hook interface

**Figure 5:**
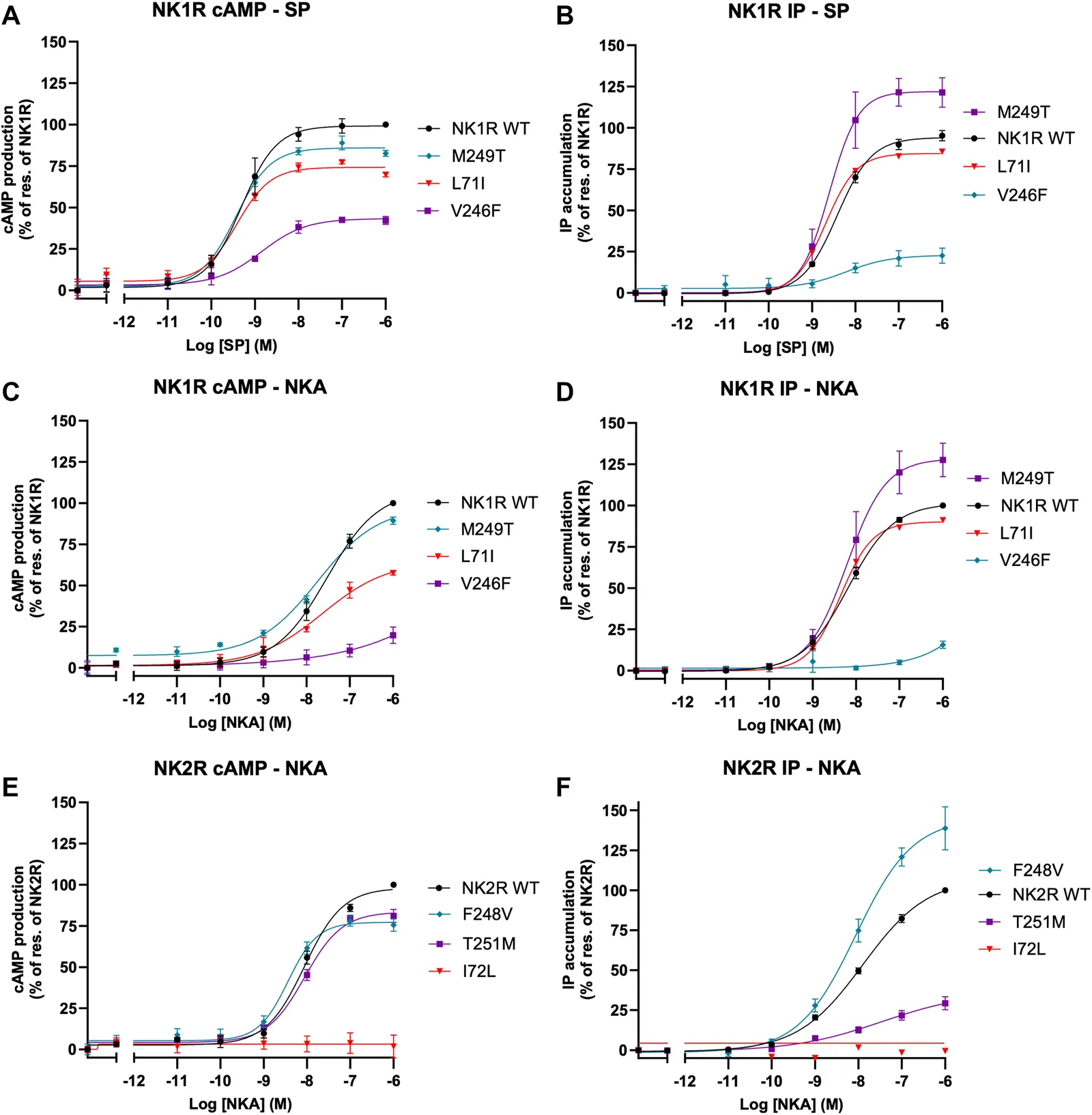
**Activation of single point NKR mutations in wavy-hook interface by SP and NKA.** BRET-based cAMP (G_s_) (*A*, *C*, and *E*) and IP_3_ accumulation (G_q_) (*B*, *D*, and *F*) responses to SP and NKA are shown. (*A*, *B*, *C*, and *D*) NK1R mutations and (*E* and *F*) NK2R mutations. Dose–response values are presented as the mean of biological replicates, with error bars indicating the SEM. Error bars were not calculated for experiments with fewer than three biological replicates. Relevant data for each graph, numbers of biological and technical replicates are provided in Table S2. The leftmost data point in each graph corresponds to the vehicle control, *i.e.* an experiment performed in the absence of ligand. Mutations are specified to the right of the graphs in the order as the corresponding graphs. BRET, bioluminescence resonance energy transfer; NK1R, NK1 receptor; NK2R, NK2 receptor; NKA, neurokinin A; NKR, neurokinin receptor; SP, substance P.

**Figure 6:**
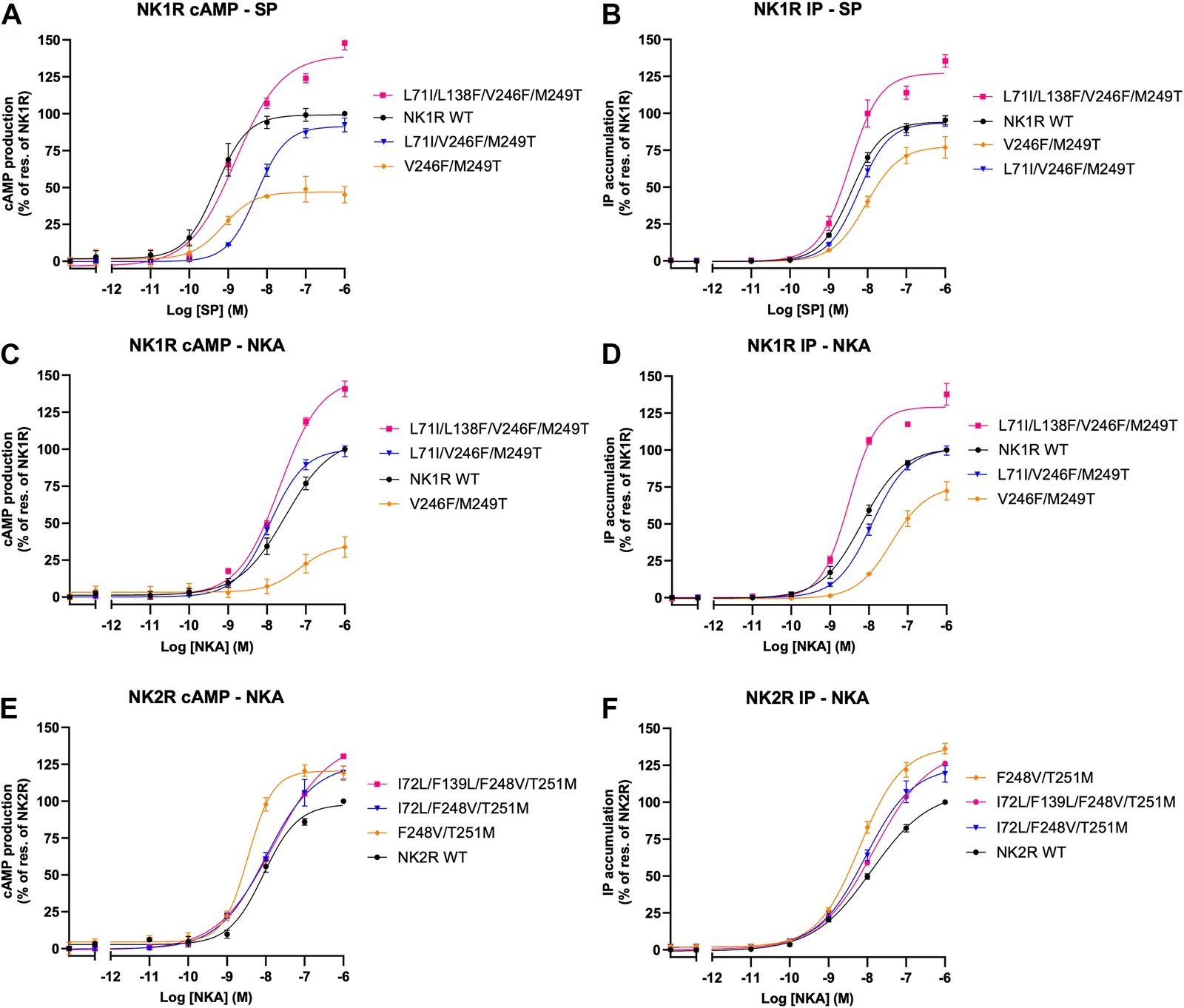
**Activation of multiple NKR point mutations in wavy-hook interface by SP and NKA.** BRET-based cAMP (G_s_) (*A*, *C*, and *E*) and IP_3_ accumulation (G_q_) (*B*, *D*, and *F*) responses to SP and NKA are shown. (*A*, *B*, *C*, and *D*) NK1R mutations and (*E* and *F*) NK2R mutations. Dose–response values are presented as the mean of biological replicates, with error bars indicating the SEM. Error bars were not calculated for experiments with fewer than three biological replicates. Relevant data for each graph, numbers of biological and technical replicates are provided in Table S3. The leftmost data point in each graph corresponds to the vehicle control, *i.e.* an experiment performed in the absence of ligand. Mutations are specified to the right of the graphs in the order as the corresponding graphs. BRET, bioluminescence resonance energy transfer; NK1R, NK1 receptor; NK2R, NK2 receptor; NKA, neurokinin A; SP, substance P.

Figures 5 and 6 present the results of reciprocal residue swapping between NK1R and NK2R. The signaling properties of the mutants were evaluated using BRET-based cAMP (G_s_) and IP_3_ accumulation (G_q_) assays in response to both SP and NKA. Figure 5 shows data for single-point mutants, whereas Figure 6 shows data for mutants containing multiple substitutions. In both figures, panels A–D correspond to NK1R mutants, while panels E–F correspond to NK2R mutants.

NK1R mutations display a clear divergence between G_s_ and G_q_ signaling. In Figure 5, *A* and *C* (G_s_ activation), mutants with enlarged side chain sizes exhibit reduced potency and efficacy compared with the WT receptor, whereas NK1R-M249^6.36^T, which has a reduced side chain size, retains NK1R-like potency with increased efficacy. In contrast, in Figure 5, *B* and *D* (G_q_ activation), NK1R-M249^6.36^T exhibits enhanced potency and efficacy. This behavior is consistent with a role of position 6.36 in modulating G-protein subtype selectivity through differential engagement of the Gα C-terminal wavy-hook. The only difference at the extreme C terminus between G_s_ and G_q_ is the residue highlighted in Figure 4 (Leu in G_s_ *versus* Val in G_q_). Substitution of NK1R-M249^6.36^ with Thr reduces side chain bulk at the receptor interface, which is expected to selectively favor tighter packing with the smaller Val at the G_q_ α5 C terminus, while maintaining compatibility with the larger Leu in Gs. Thus, residue 6.36 appears to act as a steric tuning element that differentially stabilizes receptor–G-protein complexes.

Figure 6 presents the effects of SP- and NKA-induced activation on multiple NK1R mutants. The double mutant NK1R-V246^6.33^F/M249^6.36^T exhibits lowered or negligible G_s_ activation in response to both SP and NKA (Fig. 6, *A* and *C*), whereas the corresponding reduction in G_q_ activation is less pronounced (Fig. 6, *B* and *D*).

Notably, adding the mutation NK1R-L71^2.43^ I to generate the triple mutant NK1R-L71^2.43^I/V246^6.33^F/M249^6.36^T displays differential responses to SP in G_s_ *versus* G_q_ signaling. Specifically, SP-induced G_s_ activation (Fig. 6*A*) shows a more than tenfold decrease in potency compared to NK1R, while G_q_ activation (Fig. 6*B*) remains comparable to that of the WT receptor. In contrast, NKA-induced activation of the triple mutant is similar to NK1R for both G_s_ and G_q_ (Fig. 6, *C* and *D*), differing from the behavior observed with the double mutant. Collectively, this subtle Leu-to-Ile substitution exerts a significant effect on signaling bias and modulates G_s_ and G_q_ activation in response to SP and NKA.

#### Mutagenesis of residues at the NK2R/wavy-hook interface

The results obtained for single point NK2R mutants (Fig. 5, *E* and *F*) reveal a markedly different behavior compared to the corresponding mutations in NK1R. ELISA assays (Fig. S4*B*) confirm that expression levels of NK2R and its mutants are comparable, indicating that differences in efficacy are not attributable to variations in expression levels.

Apart from NK2R-F248^6.33^V, individual point mutations reduce activity in both G_q_ and G_s_ signaling pathways. Notably, the NK2R-I72^2.43^L mutation results in complete loss of activity in both assays, underscoring the critical role of this position in both NK1R (see above) and NK2R. The mutations NK2R-F248^6.33^V and NK2R-T251^6.36^M display distinct activation profiles. In BRET-based cAMP (G_s_) assays (Fig. 5*E*) both mutants retain WT-like potency but exhibit reduced efficacy. In contrast, in IP_3_ accumulation (G_q_) assays (Fig. 5*F*), NK2R-T251^6.36^M shows negligible activity, whereas NK2R-F248^6.33^V displays enhanced potency and efficacy. These findings indicate residue 6.33 in NK2R plays a role analogous to that of position 6.36 in NK1R in modulating G_q_ signaling. Strikingly, however, the combined double mutant NK2R-F248^6.33^V/NK2R-T251^6.36^M (Fig. 6, *E* and *F*) displays a substantial increase in both potency and efficacy for G_s_ and G_q_ activation relative to WT NK2R despite the divergent profiles of the individual mutations. This contrasts with the corresponding mutations in NK1R, where activation is substantially reduced compared to the WT receptor. Adding the mutation NK2R-I72^2.43^L to obtain the triple mutant NK2R-I72^2.43^L/F248^6.33^V/NK2R-T251^6.36^M, a similar, though less pronounced, enhancement is observed as for the double mutant. Notably, the effect of introducing NK2R-I72^2.43^L into the double mutant background is particularly intriguing, as the NK2R-I72^2.43^L mutation alone results in a complete loss of activity. This nonadditive behavior suggests cooperativity between residues at positions 2.43 and 6.33/6.36, consistent with a concerted reorganization of the receptor–G-protein interface.

### Combined ICL2 and wavy–hook interaction modules in NK1R and NK2R

In this section, the combined effects of mutations spanning the ICL2 module and the wavy–hook interaction module are assessed by introducing mutations in position 138(139)^34.51^ in combination with mutants in positions 71(72)^2.43^, 246(248)^6.33^ and 249(251)^6.36^ in NK1R(NK2R).

#### ICL2 and wavy–hook interaction modules in NK1R

Figure 6, *A*–*D* show the activity profiles of combined NK1R mutants targeting the ICL2 and wavy–hook interaction modules. BRET-based cAMP (G_s_) responses to SP and NKA are presented in Figure 6, *A* and *C*, respectively, whereas IP_3_ accumulation (G_q_) responses are shown in Figure 6, *B* and *D*. In the BRET-based cAMP assay, the combined NK1R mutant (NK1R-L71^2.43^I/L138F/V246^6.33^F/M249^6.36^T) displays pronounced ligand-dependent signaling behavior.

Introduction of the NK1R-L138F mutation into the triple mutant NK1R-L71^2.43^I/V246^6.33^F/M249^6.36^T increases efficacy in response to both SP and NKA while restoring SP potency to that of the WT receptor (Fig. 6, *A* and *C*).

In the IP_3_ accumulation (G_q_) assays (Fig. 6, *B* and *D*), combining the ICL2 and wavy–hook interaction modules produces pronounced enhancements in both potency and efficacy.

#### ICL2 and wavy–hook interaction modules in NK2R

Combining the two NK2R modules to generate the mutant NK2R-I72^2.43^L/F139L/F248^6.33^V/NK2R-T251^6.36^M yields a signaling profile (Fig. 6, *E* and *F*) that differs markedly from that of the corresponding NK1R mutant (Fig. 6, *A*–*D*). In the BRET-based cAMP assay (Fig. 6*E*), NKA-induced activation displays WT-like potency but increased efficacy. In contrast, in the IP_3_ accumulation assay (Fig. 6*F*), both potency and efficacy are slightly enhanced.

The data suggest that the ICL2 and wavy-hook interface act cooperatively to stabilize productive receptor–G-protein complexes. Whereas mutations within either module alone produce receptor-specific effects, combining mutations often results in responses that cannot be predicted from the individual substitutions, indicating energetic coupling between the two intracellular interaction networks.

## Conclusions

This study identifies two cooperative intracellular structural modules that together govern G-protein coupling and signaling bias in the tachykinin receptors NK1R and NK2R. The first module comprises the unusually short, proline-rich ICL2, which adopts a conformationally strained active-state geometry that is essential for productive G-protein engagement. Sequence analysis, MD simulations, and mutagenesis collectively demonstrate that this conserved PxxPRL motif imposes structural constraints that shape the active ICL2 conformation and stabilize productive receptor–G-protein interactions. These findings provide a structural rationale for the lack of constitutive activity observed in tachykinin receptors, as receptor activation requires overcoming the energetic penalty associated with formation of the active ICL2 conformation.

The second module is formed by the intracellular receptor surface that accommodates the C-terminal α5 helix (wavy-hook) of Gα. Although the overall receptor–G-protein interfaces of NK1R and NK2R are highly conserved, reciprocal mutagenesis reveals that a limited number of nonconserved residues at positions 2.43, 6.33, and 6.36 are sufficient to alter G-protein coupling efficiency and signaling bias. In particular, residue 6.36 in NK1R and residue 6.33 in NK2R act as steric tuning elements that differentially modulate G_s_ and G_q_ coupling, demonstrating how subtle changes in intracellular pocket chemistry influence G-protein subtype selectivity.

Importantly, the ICL2 and wavy-hook modules do not function independently. Rather, combining mutations in both modules produces nonadditive effects on signaling, indicating functional cooperativity between the two modules. Together, these findings establish that G-protein selectivity in tachykinin receptors is governed by the interplay between a conformationally constrained ICL2 and a tunable wavy-hook interface. More broadly, they demonstrate that relatively small alterations in intracellular receptor architecture can reprogram signaling bias without directly affecting ligand recognition, providing a mechanistic framework for understanding and engineering G-protein selectivity in class A GPCRs.

Interestingly, the nature of this cooperativity differs between NK1R and NK2R. In NK1R, combining ICL2 and wavy-hook mutations largely restores or enhances signaling, whereas in NK2R the same mutational strategy produces distinct functional outcomes despite the high sequence similarity of the two receptors. These observations indicate that the energetic coupling between the two intracellular modules is receptor-specific and contributes to subtype-dependent regulation of G-protein coupling.

Collectively, these findings establish the ICL2 and wavy-hook interface as a coupled intracellular signaling network that controls G-protein selectivity in tachykinin receptors and provides a mechanistic framework for understanding and engineering biased signaling in class A GPCRs.

## Experimental procedures

### Structural modeling

Structural visualization was performed in PyMOL (open source version 2.5.0, Schrödinger LLC, http://www.pymol.org/pymol).

### MD simulations

*Gromacs/CHARMM36m forcefield*: Input files for the MD simulations of the four models of NK1R complexes and NK1R apo were from PDB ID:7P00 and PDB:6hlp modeled in PyMOL and prepared in CHARMM-GUI (42, 43). The two complexes of NK2R: NK2R/NKA and NK2R/NKA/miniGq were from PDB ID:7XWO. The standard input generator for membrane builder (bilayer builder) was used. The position of the membrane bilayer was calculated using the PPM server (44). The system was solvated using 24,550TIP3P waters (45) in a rectangular box (90 × 90 × 135 Å^3^). Ionic strength of 150 mM was obtained by adding Cl^-^ and Na^+^ ions and the membrane was composed of DOPS lipids. The simulations were performed at 303 K using a Nosé-Hoover thermostat in an NPT (constant pressure using Parrinello-Rahman barostat (46) ensemble using Gromacs (47) and the CHARMM36m force field (45). The system was minimized and simulated for more than 2000 ns. The trajectory was analyzed using Gromacs tools and in-house scripts. Plotting was done using Grace (xmgrace; https://plasma-gate.weizmann.ac.il/Grace/) or matplotlib (48).

### Compounds

Compounds were dissolved in 100% dimethyl sulfoxide or 70% ethanol. NKA and SP were from Sigma-Aldrich (N4267 and S6883, respectively). Peptide purity exceeding 98%.

### Plasmids

All receptor constructs of WT and modified human *TACR1* and *TACR2* were inserted into the pcDNA3.1(+)-C-DYK vector (Genscript) whereas CAMYEL (49) was expressed *via* pcDNA3.1(+) vector.

### Cell culture, plating and transfection

Cos7 cell line was purchased from American Type Culture Collection (CRL-1651). COS cells were chosen because they typically produce higher levels of recombinant protein and exhibit greater transfection efficiency compared to HEK cells. In addition, COS cells are well-suited for generating stable cell lines and are sometimes more effective at expressing and secreting membrane-bound proteins. COS7 cells were maintained in Dulbecco´s Modified Eagle´s Medium 1885 with GlutaMAX supplemented with 10% fetal bovine serum (Sigma-Aldrich), 100 units/ml penicillin and 100 μg/ml streptomycin at 37 °C with 10% CO_2_. COS7 cells were tested monthly for *mycoplasma* and kept negative. COS7 cells were plated in either clear- or white- or white with clear bottom 96-well plates that were coated with poly-D-lysine (Sigma-Aldrich) 30 min prior to cell seeding (20.000 cells/well). The following day plates were transiently transfected using calcium precipitation with 200 ng/well DNA of receptor DNA and for the cAMP assays a 1000 ng/well of CAMYEL in a culture medium with the addition of 100 μM (final concentration) Chloroquine. Transfection was stopped 5 h later with the addition of a fresh maintenance medium.

### BRET-based cAMP assay

The intracellular level of cAMP was monitored in real-time using BRET. This was achieved by implementing a construct consisting of a cAMP binding protein (exchange protein activated by cAMP (EPAC)) which has been flanked by a BRET pair consisting of Renilla luciferase (Rluc) and YFP. This construct is called CAMYEL (cAMP sensor using YFP-Epac-Rluc) and enables cAMP production to be sensed as Epac changes conformation in response to increasing levels of cAMP, ultimately resulting in a loss of BRET signal.

On the day of the assay, white 96-well plates with COS7 cells were washed twice with 100 μl/well Hank’s Balanced Salt Solution (HBSS) (GIBCO, Life Technologies) and preincubated for 30 min at 37 °C with 85 μl HBSS. Luciferase substrate coelenterazine h (Thermo Fisher Scientific) was added to a final concentration of 5 μM and a baseline measurement was taken after 5 min. Dose-response curves of either NKA or SP were added and measurements were recorded every minute for 30 min on a CLARIOstar Plus plate reader. The BRET signal was calculated as the ratio of emission intensity at 535 nm (citrine) to the emission intensity at 475 nm (luciferase). Determinations were made in minimum duplicates.

### IP_3_ accumulation assay

COS7 cells seeded in clear 96-well plates were incubated with 0.5 μCi/ml myo [3H]inositol (PerkinElmer) in 100 μl growth medium overnight following the transfection. The subsequent day cells were washed twice with 200 μl/well HBSS (GIBCO, Life Technologies) and preincubated for 5 min at 37 °C with 100 μl/well HBSS buffer supplemented with 10 mM LiCl. Ligand addition was followed by 90 min incubation at 37 °C. To stop ligand incubation, cells were lysed with 40 μl 10 mM formic acid followed by 30 min incubation on ice. A total of 35 μl of the lysate was transferred to white 96-well plates together with 60 μl of 1:8 diluted YSi SPA scintillation beads (PerkinElmer). Plates were sealed and vigorously shaken for 15 min followed by 5 min centrifugation at 1500 rpm. Measurements (scintillation) were recorded on a Microbeta (PerkinElmer) after a 4 h delay and determinations were made in duplicates.

### Statistical analysis

All dose-response curves have been calculated using Prism 10 software (GraphPad Software, www.graphpad.com) nonlinear regression with four parameters. All data are shown as mean ± SEM unless stated otherwise and consist of three biological replicates from two technical replicates.

## Data availability

MD trajectories corresponding to the data presented in Figs. S1 and S2*B*, *C* have been uploaded to Zenodo (https://doi.org/10.5281/zenodo.14512995).

## Supporting information

This article contains supporting information.

## Conflict of interest

The authors declare that they have no conflicts of interest with the contents of this article.
