# Supplementary material for: Intracellular pocket and ICL2 motifs govern G-protein coupling bias in NK1 receptor (TACR1) and NK2 receptor (TACR2)
Source: J Biol Chem. 2026 Aug 12;302(10):113437. doi: 10.1016/j.jbc.2026.113437 (PMC13570342; doi:10.1016/j.jbc.2026.113437)
Supplement: Supplementary file 1 [file mmc1.docx]

**Supporting Information**

**Intracellular pocket and ICL2 motifs govern G-protein coupling bias in NK_1_ receptor (TACR1) and NK_2_ receptor (TACR2)**

Jacob E. Petersen^1^, Artem Pavlovskyi^1^, Jesper J. Madsen^2,3^, Thue W. Schwartz^1^, Thomas M. Frimurer^1^, and Ole H. Olsen^1*^

^1^ Novo Nordisk Foundation Center for Basic Metabolic Research, University of Copenhagen, Blegdamsvej 3b, DK-2200 Copenhagen N, Denmark

^2^ Department of Molecular Medicine, Morsani College of Medicine, University of South Florida, Tampa, Florida 33612, United States of America

^3^ Center for Global Health and Infectious Diseases Research, Global and Planetary Health, College of Public Health, University of South Florida, Tampa, Florida 33612, United States of America

^*^Correspondence: Ole H. Olsen, Novo Nordisk Foundation Center for Basic Metabolic Research, University of Copenhagen, Blegdamsvej 3b, DK-2200 Copenhagen N, Denmark. Tel.: +45 60661288. E-mail: [oho@sund.ku.dk](mailto:oho@sund.ku.dk)

This document contains four figures and three tables .

Figure S1. ICL2 sequences. NKRs have a short ICL2 loop with an unusual proline motif. (A) ICL2 length distribution in class A GPCRs from GPCRdb (N = 312). Neurokinin receptors stand out from the most common ICL2 length of 8 by having a loop length of only 6. (B) Proline spacing analysis for class A GPCR ICL2 sequences with poly-proline motifs. k denotes the number of residues between two prolines: PP = 0, PxP = 1, PxxP = 2, etc. Purple points indicate motifs with exactly two prolines, and therefore one k value. Yellow points indicate motifs with three prolines, which gives three pairwise k spacing values; connected labeled points mark these cases (gpbar_human, ccr10_human, and gpr62_human). (C) Sequence logo of class A GPCR ICL2 sequences with length 6 and a PxxP motif. (D) ICL2 sequences of length 6 from GPCRs, annotated with receptor names, all containing a PxxP motif.

**Figure S2. MD simulations, RMSD vs time.** RMSD values for the upper and lower halves of the transmembrane (TM) bundle are shown in black and red, respectively (TMupper and TMlower), with running averages indicated in white. TMupper is defined by residue ranges 30-45, 81-94, 102-118, 156-168, 191-205, 256-273 and 283-295 and TMlower by residue ranges 46-58, 67-80, 119-135,144-155, 206-224, 241-255 and 296-308. (A) NK1R in complex with the high-affinity antagonist netupitant, (PDB: 6HLP), (B) an apo NK1R model derived from 6HLP with netupitant removed, (C) an NK1R structure with SP bound (based on PDB: 7P00 with miniGq removed), and (D) the intact 7P00 structure.

**Figure S3. φ/ψ distributions of ICL2 residues in NK2R.** Ramachandran maps of Pro138 (black), Phe139 (red), and Pro141 (green) from simulations of NK2R/NKA, and NK2R/NKA/miniG_q_. The lower panel shows the φ/ψ distribution of Phe139.

**Figure S4. Results from ELISA assays.** (A) Comparison of expression levels for NK1R and its mutants discussed in the text. (B) Comparison of expression levels for NK2R and its mutants discussed in the text.

|  | Mutants | cAMP | | | | IP3 | | | |
| --- | --- | --- | --- | --- | --- | --- | --- | --- | --- |
|  |  | log(EC_50_) ± SD | E_max_  (% of WT response) | Biological repeats | Technical repeats | log(EC_50_) ± SD | E_max_  (% of WT response) | Biological repeats | Technical repeats |
| SP | NK1R | -9.2 ± 0.04 | 100 | 3 | 2 | -9.0 ± 0.08 | 100 | 3 | 2 |
|  | NK1R P137A^ICL2^ | N/A | 6 | 3 | 2 | N/A | 4 | 4 | 2 |
|  | NK1R P137V^ICL2^ | N/A | 0 | 3 | 2 | -9.0 ± 0.51 | 34 | 4 | 2 |
|  | NK1R L138A^ICL2^ | -9.1 ± 0.24 | 20 | 3 | 2 | N/A | 0 | 3 | 2 |
|  | NK1R L138F^ICL2^ | -9.5 ± 0.11 | 120 | 3 | 2 | -8.6 ± 0.08 | 142 | 3 | 2 |
|  | NK1R P140A^ICL2^ | -8.9 ± 0.09 | 98 | 3 | 2 | -8.7 ± 0.04 | 76 | 3 | 2 |
|  | NK1R P140V^ICL2^ | -8.8 ± 0.12 | 85 | 3 | 2 | -8.4 ± 0.06 | 84 | 3 | 2 |
|  | NK1R P137A^ICL2^/P140V^ICL2^ | N/A | 0 | 3 | 2 | N/A | 0 | 3 | 2 |
| NKA | NK1R | -7.5 ± 0.05 | 100 | 3 | 2 | -8.3 ± 0.12 | 100 | 3 | 2 |
|  | NK1R P137A^ICL2^ | N/A | 0 | 3 | 2 | N/A | 6 | 4 | 2 |
|  | NK1R P137V^ICL2^ | N/A | 4 | 3 | 2 | -8.2 ± 0.34 | 40 | 4 | 2 |
|  | NK1R L138A^ICL2^ | -7.3 ± 0.46 | 16 | 3 | 2 | N/A | 0 | 3 | 2 |
|  | NK1R L138F^ICL2^ | -8.3 ± 0.08 | 130 | 3 | 2 | -8.4 ± 0.08 | 140 | 3 | 2 |
|  | NK1R P140A^ICL2^ | -7.2 ± 0.09 | 90 | 3 | 2 | -8.2 ± 0.05 | 85 | 3 | 2 |
|  | NK1R P140V^ICL2^ | -7.1 ± 0.11 | 68 | 3 | 2 | -8.0 + 0.04 | 88 | 3 | 2 |
|  | NK1R P137A^ICL2^/P140V^ICL2^ | N/A | 0 | 3 | 2 | N/A | 0 | 3 | 2 |
|  | NK2R | -8.0 ± 0.08 | 100 | 3 | 2 | -7.9 ± 0.06 | 100 | 3 | 2 |
|  | NK2R F139A^ICL2^ | N/A | 0 | 3 | 2 | N/A | 7 | 2 | 2 |
|  | NK2R F139L^ICL2^ | -8.0 ± 0.12 | 124 | 3 | 2 | -8.0* | 124 | 2 | 2 |

*Statistics were not performed due to number of biological repeats being less than 2.

**Table S1. Data corresponding to the graphs shown in Figure 3.**

|  | Mutants | cAMP | | | | IP3 | | | |
| --- | --- | --- | --- | --- | --- | --- | --- | --- | --- |
|  |  | log(EC_50_) ± SD | E_max_  (% of WT response) | Biological repeats | Technical repeats | log(EC_50_) ± SD | E_max_  (% of WT response) | Biological repeats | Technical repeats |
| SP | NK1R WT | -9.3 ± 0.16 | 100 | 3 | 2 | -8.3 ± 0.08 | 100 | 4 | 2 |
|  | NK1R L71I^2.43^ | -9.5 ± 0.18 | 70 | 3 | 2 | -8,7** | 85 | 2 | 2 |
|  | NK1R V246F^6.33^ | -8.9 ± 0.29 | 42 | 3 | 2 | -8.3 ± 0.55 | 22 | 3 | 2 |
|  | NK1R M249T^6.36^ | -9.5 ± 0.11 | 83 | 3 | 2 | -8.6 ± 0.17 | 121 | 3 | 2 |
| NKA | NK1R WT | -7.6 ± 0.10 | 100 | 3 | 2 | -8.2 ± 0.09 | 100 | 5 | 2 |
|  | NK1R L71I^2.43^ | -7.8 ± 0.21 | 57* | 3 | 2 | -8.3** | 91 | 2 | 2 |
|  | NK1R V246F^6.33^ | ≥ -6.5 | 20* | 3 | 2 | ≥ -6.5 | 16* | 4 | 2 |
|  | NK1R M249T^6.36^ | -7.9 ± 0.13 | 89 | 3 | 2 | -8.2 ± 0.18 | 128 | 3 | 2 |
|  | NK2R WT | -8.0 ± 0.08 | 100 | 3 | 2 | -8.0 ± 0,07 | 100 | 4 | 2 |
|  | NK2R I72L^2.43^ | N/A | 0 | 3 | 2 | N/A | 0 | 2 | 2 |
|  | NK2R F248V ^6.33^ | -8.5 ± 0.15 | 75 | 3 | 2 | -8.1 ± 0,17 | 139 | 3 | 2 |
|  | NK2R T251M^6.36^ | -8.1 ± 0.09 | 81 | 3 | 2 | -7.9 ± 0.33 | 29* | 3 | 2 |

*Emax was not reached, values presented at agonist concentration of −6 of logarithmic scale.

**Statistics were not performed due to number of biological repeats being less than 2.

**Table S2. Data corresponding to the graphs shown in Figure 5.**

|  | Mutants | cAMP | | | | IP3 | | | |
| --- | --- | --- | --- | --- | --- | --- | --- | --- | --- |
|  |  | log(EC_50_) ± SD | E_max_  (% of WT response) | Biological repeats | Technical repeats | log(EC_50_) ± SD | E_max_  (% of WT response) | Biological repeats | Technical repeats |
| SP | NK1R WT | -9.3 ± 0.16 | 100 | 3 | 2 | -8.3 ± 0.08 | 100 | 4 | 2 |
|  | NK1R V246F^6.33^, M249T^6.36^ | -9.2 ± 0.30 | 45 | 3 | 2 | -8.0 ± 0.11 | 77 | 3 | 2 |
|  | NK1R L71I^2.43^, V246F^6.33^, M249T^6.36^ | -8.3 ± 0.09 | 92 | 4 | 2 | -8.2 ± 0.07 | 95 | 4 | 2 |
|  | NK1R L71I^2.43^, L138F^ICL2^, V246F^6.33^, M249T^6.36^ | -8.7 ± 0.15 | 148 | 3 | 2 | -8.4 ± 0.12 | 135 | 3 | 2 |
| NKA | NK1R WT | -7.6 ± 0.10 | 100 | 3 | 2 | -8.2 ± 0.09 | 100 | 5 | 2 |
|  | NK1R V246F^6.33^, M249T^6.36^ | -7.2 ± 0.37 | 34 | 3 | 2 | -7.5 ± 0.10 | 72 | 3 | 2 |
|  | NK1R L71I^2.43^, V246F^6.33^, M249T^6.36^ | -7.9 ± 0.07 | 99 | 4 | 2 | -7.9 ± 0.06 | 100 | 4 | 2 |
|  | NK1R L71I^2.43^, L138F^ICL2^, V246F^6.33^, M249T^6.36^ | -7.7 ± 0.07 | 141 | 3 | 2 | -8.4 ± 0.10 | 138 | 3 | 2 |
|  | NK2R WT | -8.0 ± 0.08 | 100 | 3 | 2 | -8.0 ± 0,07 | 100 | 4 | 2 |
|  | NK2R F248V ^6.33^, T251M^6.36^ | -8.5 ± 0.09 | 120 | 3 | 2 | -8.2 ± 0.06 | 136 | 3 | 2 |
|  | NK2R I72L^2.43^, F248V ^6.33^, T251M^6.36^ | -8.1 ± 0.11 | 119 | 3 | 2 | -8.1 ± 0.10 | 119 | 3 | 2 |
|  | NK2R I72L^2.43^, F139L^ICL2^, F248V ^6.33^, T251M^6.36^ | -7.9 ± 0.08 | 130 | 3 | 2 | -7.9 ± 0.08 | 126 | 3 | 2 |

**Table S3. Data corresponding to the graphs shown in Figure 6.**
