# Supplementary material for: Intracellular pocket and ICL2 motifs govern G-protein coupling bias in NK1 receptor (TACR1) and NK2 receptor (TACR2)
Source: J Biol Chem. 2026 Aug 12;302(10):113437. doi: 10.1016/j.jbc.2026.113437 (PMC13570342; doi:10.1016/j.jbc.2026.113437)
Supplement: Supplementary file 2 [file mmc2.docx]

|  | Mutants | cAMP | | | | IP3 | | | |
| --- | --- | --- | --- | --- | --- | --- | --- | --- | --- |
|  |  | log(EC_50_) ± SD | E_max_  (% of WT response) | Biological repeats | Technical repeats | log(EC_50_) ± SD | E_max_  (% of WT response) | Biological repeats | Technical repeats |
| SP | NK1R | -9.2 ± 0.04 | 100 | 3 | 2 | -9.0 ± 0.08 | 100 | 3 | 2 |
|  | NK1R P137A^ICL2^ | N/A | 6 | 3 | 2 | N/A | 4 | 4 | 2 |
|  | NK1R P137V^ICL2^ | N/A | 0 | 3 | 2 | -9.0 ± 0.51 | 34 | 4 | 2 |
|  | NK1R L138A^ICL2^ | -9.1 ± 0.24 | 20 | 3 | 2 | N/A | 0 | 3 | 2 |
|  | NK1R L138F^ICL2^ | -9.5 ± 0.11 | 120 | 3 | 2 | -8.6 ± 0.08 | 142 | 3 | 2 |
|  | NK1R P140A^ICL2^ | -8.9 ± 0.09 | 98 | 3 | 2 | -8.7 ± 0.04 | 76 | 3 | 2 |
|  | NK1R P140V^ICL2^ | -8.8 ± 0.12 | 85 | 3 | 2 | -8.4 ± 0.06 | 84 | 3 | 2 |
|  | NK1R P137A^ICL2^/P140V^ICL2^ | N/A | 0 | 3 | 2 | N/A | 0 | 3 | 2 |
| NKA | NK1R | -7.5 ± 0.05 | 100 | 3 | 2 | -8.3 ± 0.12 | 100 | 3 | 2 |
|  | NK1R P137A^ICL2^ | N/A | 0 | 3 | 2 | N/A | 6 | 4 | 2 |
|  | NK1R P137V^ICL2^ | N/A | 4 | 3 | 2 | -8.2 ± 0.34 | 40 | 4 | 2 |
|  | NK1R L138A^ICL2^ | -7.3 ± 0.46 | 16 | 3 | 2 | N/A | 0 | 3 | 2 |
|  | NK1R L138F^ICL2^ | -8.3 ± 0.08 | 130 | 3 | 2 | -8.4 ± 0.08 | 140 | 3 | 2 |
|  | NK1R P140A^ICL2^ | -7.2 ± 0.09 | 90 | 3 | 2 | -8.2 ± 0.05 | 85 | 3 | 2 |
|  | NK1R P140V^ICL2^ | -7.1 ± 0.11 | 68 | 3 | 2 | -8.0 + 0.04 | 88 | 3 | 2 |
|  | NK1R P137A^ICL2^/P140V^ICL2^ | N/A | 0 | 3 | 2 | N/A | 0 | 3 | 2 |
|  | NK2R | -8.0 ± 0.08 | 100 | 3 | 2 | -7.9 ± 0.06 | 100 | 3 | 2 |
|  | NK2R F139A^ICL2^ | N/A | 0 | 3 | 2 | N/A | 7 | 2 | 2 |
|  | NK2R F139L^ICL2^ | -8.0 ± 0.12 | 124 | 3 | 2 | -8.0* | 124 | 2 | 2 |

*Statistics were not performed due to number of biological repeats being less than 2.
