# Supplementary material for: Intracellular pocket and ICL2 motifs govern G-protein coupling bias in NK1 receptor (TACR1) and NK2 receptor (TACR2)
Source: J Biol Chem. 2026 Aug 12;302(10):113437. doi: 10.1016/j.jbc.2026.113437 (PMC13570342; doi:10.1016/j.jbc.2026.113437)
Supplement: Supplementary file 3 [file mmc3.docx]

|  | Mutants | cAMP | | | | IP3 | | | |
| --- | --- | --- | --- | --- | --- | --- | --- | --- | --- |
|  |  | log(EC_50_) ± SD | E_max_  (% of WT response) | Biological repeats | Technical repeats | log(EC_50_) ± SD | E_max_  (% of WT response) | Biological repeats | Technical repeats |
| SP | NK1R WT | -9.3 ± 0.16 | 100 | 3 | 2 | -8.3 ± 0.08 | 100 | 4 | 2 |
|  | NK1R L71I^2.43^ | -9.5 ± 0.18 | 70 | 3 | 2 | -8,7** | 85 | 2 | 2 |
|  | NK1R V246F^6.33^ | -8.9 ± 0.29 | 42 | 3 | 2 | -8.3 ± 0.55 | 22 | 3 | 2 |
|  | NK1R M249T^6.36^ | -9.5 ± 0.11 | 83 | 3 | 2 | -8.6 ± 0.17 | 121 | 3 | 2 |
| NKA | NK1R WT | -7.6 ± 0.10 | 100 | 3 | 2 | -8.2 ± 0.09 | 100 | 5 | 2 |
|  | NK1R L71I^2.43^ | -7.8 ± 0.21 | 57* | 3 | 2 | -8.3** | 91 | 2 | 2 |
|  | NK1R V246F^6.33^ | ≥ -6.5 | 20* | 3 | 2 | ≥ -6.5 | 16* | 4 | 2 |
|  | NK1R M249T^6.36^ | -7.9 ± 0.13 | 89 | 3 | 2 | -8.2 ± 0.18 | 128 | 3 | 2 |
|  | NK2R WT | -8.0 ± 0.08 | 100 | 3 | 2 | -8.0 ± 0,07 | 100 | 4 | 2 |
|  | NK2R I72L^2.43^ | N/A | 0 | 3 | 2 | N/A | 0 | 2 | 2 |
|  | NK2R F248V ^6.33^ | -8.5 ± 0.15 | 75 | 3 | 2 | -8.1 ± 0,17 | 139 | 3 | 2 |
|  | NK2R T251M^6.36^ | -8.1 ± 0.09 | 81 | 3 | 2 | -7.9 ± 0.33 | 29* | 3 | 2 |

*Emax was not reached, values presented at agonist concentration of −6 of logarithmic scale.

**Statistics were not performed due to number of biological repeats being less than 2.
