# Supplementary material for: Intracellular pocket and ICL2 motifs govern G-protein coupling bias in NK1 receptor (TACR1) and NK2 receptor (TACR2)
Source: J Biol Chem. 2026 Aug 12;302(10):113437. doi: 10.1016/j.jbc.2026.113437 (PMC13570342; doi:10.1016/j.jbc.2026.113437)
Supplement: Supplementary file 4 [file mmc4.docx]

|  | Mutants | cAMP | | | | IP3 | | | |
| --- | --- | --- | --- | --- | --- | --- | --- | --- | --- |
|  |  | log(EC_50_) ± SD | E_max_  (% of WT response) | Biological repeats | Technical repeats | log(EC_50_) ± SD | E_max_  (% of WT response) | Biological repeats | Technical repeats |
| SP | NK1R WT | -9.3 ± 0.16 | 100 | 3 | 2 | -8.3 ± 0.08 | 100 | 4 | 2 |
|  | NK1R V246F^6.33^, M249T^6.36^ | -9.2 ± 0.30 | 45 | 3 | 2 | -8.0 ± 0.11 | 77 | 3 | 2 |
|  | NK1R L71I^2.43^, V246F^6.33^, M249T^6.36^ | -8.3 ± 0.09 | 92 | 4 | 2 | -8.2 ± 0.07 | 95 | 4 | 2 |
|  | NK1R L71I^2.43^, L138F^ICL2^, V246F^6.33^, M249T^6.36^ | -8.7 ± 0.15 | 148 | 3 | 2 | -8.4 ± 0.12 | 135 | 3 | 2 |
| NKA | NK1R WT | -7.6 ± 0.10 | 100 | 3 | 2 | -8.2 ± 0.09 | 100 | 5 | 2 |
|  | NK1R V246F^6.33^, M249T^6.36^ | -7.2 ± 0.37 | 34 | 3 | 2 | -7.5 ± 0.10 | 72 | 3 | 2 |
|  | NK1R L71I^2.43^, V246F^6.33^, M249T^6.36^ | -7.9 ± 0.07 | 99 | 4 | 2 | -7.9 ± 0.06 | 100 | 4 | 2 |
|  | NK1R L71I^2.43^, L138F^ICL2^, V246F^6.33^, M249T^6.36^ | -7.7 ± 0.07 | 141 | 3 | 2 | -8.4 ± 0.10 | 138 | 3 | 2 |
|  | NK2R WT | -8.0 ± 0.08 | 100 | 3 | 2 | -8.0 ± 0,07 | 100 | 4 | 2 |
|  | NK2R F248V ^6.33^, T251M^6.36^ | -8.5 ± 0.09 | 120 | 3 | 2 | -8.2 ± 0.06 | 136 | 3 | 2 |
|  | NK2R I72L^2.43^, F248V ^6.33^, T251M^6.36^ | -8.1 ± 0.11 | 119 | 3 | 2 | -8.1 ± 0.10 | 119 | 3 | 2 |
|  | NK2R I72L^2.43^, F139L^ICL2^, F248V ^6.33^, T251M^6.36^ | -7.9 ± 0.08 | 130 | 3 | 2 | -7.9 ± 0.08 | 126 | 3 | 2 |
